# Pancreatic duct ligation reduces premalignant pancreatic lesions in a Kras model of pancreatic adenocarcinoma in mice

**DOI:** 10.1038/s41598-020-74947-4

**Published:** 2020-10-27

**Authors:** Marta Cáceres, Rita Quesada, Mar Iglesias, Francisco X. Real, Maria Villamonte, Jaime Martinez de Villarreal, Mónica Pérez, Ana Andaluz, Xavier Moll, Enrique Berjano, Dimitri Dorcaratto, Patricia Sánchez-Velázquez, Luís Grande, Fernando Burdío

**Affiliations:** 1 Department of Surgery, Hospital del Mar, Barcelona, Spain; 2 Epithelial Carcinogenesis Group, Molecular Oncology Programme, Centro Nacional de Investigaciones Oncológicas, Madrid, Spain; 3 Departament de Medicina i Cirurgia Animals, Facultat de Veterinària, Universitat Autònoma de Barcelona, Barcelona, Spain; 4 BioMIT, Department of Electronic Engineering, Universitat Politècnica de València, Valencia, Spain; 5 Hospital Clínico de Valencia, Valencia, Spain

**Keywords:** Cancer, Diseases, Medical research, Pathogenesis

## Abstract

Pancreatic duct ligation (PDL) in the murine model has been described as an exocrine pancreatic atrophy-inducing procedure. However, its influence has scarcely been described on premalignant lesions. This study describes the histological changes of premalignant lesions and the gene expression in a well-defined model of pancreatic ductal adenocarcinoma by PDL. Selective ligation of the splenic lobe of the pancreas was performed in Ptf1a-Cre^(+/ki)^; K-ras LSLG12Vgeo^(+/ki)^ mice (PDL-Kras mice). Three experimental groups were evaluated: PDL group, controls and shams. The presence and number of premalignant lesions (PanIN 1–3 and Atypical Flat Lesions—AFL) in proximal (PP) and distal (DP) pancreas were studied for each group over time. Microarray analysis was performed to find differentially expressed genes (DEG) between PP and PD. Clinical human specimens after pancreaticoduodenectomy with ductal occlusion were also evaluated. PDL-Kras mice showed an intense pattern of atrophy in DP which was shrunk to a minimal portion of tissue. Mice in control and sham groups had a 7 and 10-time increase respectively of risk of high-grade PanIN 2 and 3 and AFL in their DP than PDL-Kras mice. Furthermore, PDL-Kras mice had significantly less PanIN 1 and 2 and AFL lesions in DP compared to PP. We identified 38 DEGs comparing PP and PD. Among them, several mapped to protein secretion and digestion while others such as Nupr1 have been previously associated with PanIN and PDAC. PDL in Ptf1a-Cre^(+/ki)^; K-ras LSLG12Vgeo^(+/ki)^ mice induces a decrease in the presence of premalignant lesions in the ligated DP. This could be a potential line of research of interest in some cancerous risk patients.

## Introduction

In spite of advanced basic and applied research, the prognosis of pancreatic ductal adenocarcinoma (PDAC) has not improved for the last 20 years, being the overall 5-year survival of less than 5%^1^. Only less than 20% patients are diagnosed early enough to benefit from a definitive surgical treatment^2,3^ and PDAC is projected to surpass breast, prostate, and colorectal cancers as the leading cause of cancer-related death in the US by the year 2030^2^. For those reasons, the development of the first genetically defined model of PDAC that recapitulates the morphological spectrum of the human disease was welcomed with enthusiasm. In that regard, the conditional knock-in Kras^G12D/+^ mouse model, first described by Hingorani et al.^4^ has been used in basic research to study the importance of Kras mutations in PDAC initiation. Upon activation of mutant Kras during pancreatic development, mice develop premalignant lesions starting at week 10 and this model has become the most widely used to study the evolution to PDAC^5^. Additionally, atypical flat lesions (AFL) have been described in this genetically engineered mouse model (GEMM) of adenocarcinoma and in humans as preneoplastic lesions^6–8^.

Kras mutations are one of the earliest genetic abnormalities in the progression model to PDAC^5,9^. Ninety percent of human PDAC contain activating mutations in the GTPase signaling protein—Kras oncogene, leading to PanIN-sequence initiation^10,11^. The p53 tumor suppressor gene is the central integrator of the cellular response to DNA damage and apoptosis^12,13^. Loss of p53 function causes deregulation in cell death and cell division^14,15^. Interestingly, p53 mutation, assessed by nuclear overexpression of p53 protein, is a late event in the progression model of pancreatic adenocarcinoma^5,15^ (Figure S1).

On the other hand, ultrastructural and immunohistochemical studies in normal and transgenic mice have demonstrated that after experimental pancreatic duct ligation (PDL), most of the acinar cells are rapidly and massively deleted by apoptosis-mediated exocrine atrophy. This process is also regulated by p53 active protein^13,16–19^. In this regard, our group has demonstrated a complete acinar cell deletion both in short and long-term evolution with no sign of acinar regeneration after an efficient model of PDL in murine and also in pig normal pancreas^20,21^.

Taken these pieces of evidences in mind, our hypothesis is that selective PDL in Ptf1a-Cre ^(+/ki)^; K-ras LSLG12Vgeo ^(+/ki)^ mice at an early stage (in the absence of p53 inactivation) can induce apoptotic-mediated exocrine atrophy, and may avoid progression to PDAC, overcoming pro-oncogenic stimulation. To study this, we examined the histological changes and the gene expression that take place in the distal and proximal pancreas after PDL.

## Materials and methods

### Study design

Twelve-week old Ptf1a-Cre ^(+/ki)^; K-ras LSLG12Vgeo ^(+/ki)^ mice referred as Kras mice and two control groups K-RasLSLG12Vgeo^(+/ki)^ mice and Ptf1a-Cre^(+/ki)^ mice referred to as G and Cre mice respectively^22,23^ were used in this study. All animals were bred and raised until 8–10 weeks at the Spanish National Cancer Research Center (CNIO, Madrid), then imported to Barcelona's Biomedical Research Park (PRBB) facilities. The research protocol was approved by the Ethics Committee for Animal Experimentation of PRBB (Ref.: FBP-12-1422) and the study was conducted according to the approved guidelines of the Government of Catalonia's Animal Care Committee (Ref.: DAAM 6529). All experiments were performed in accordance with relevant guidelines and regulations.

Kras mice were divided in three experimental groups: non operated animals referred to as “control group” (n = 13), animals that were operated and surgically manipulated but did not undergo pancreatic ligation referred to as “sham group” (n = 8) and animals subjected to a pancreatic duct ligation referred to as “PDL group” (n = 26). Mice were sacrificed and analyzed 1, 3 and 6 months postoperatively (PO). Additionally, G (n = 12) and Cre mice (n = 11) were also subjected to PDL and were sacrificed at the same PO periods.

### Surgical technique

**Figure 1:**
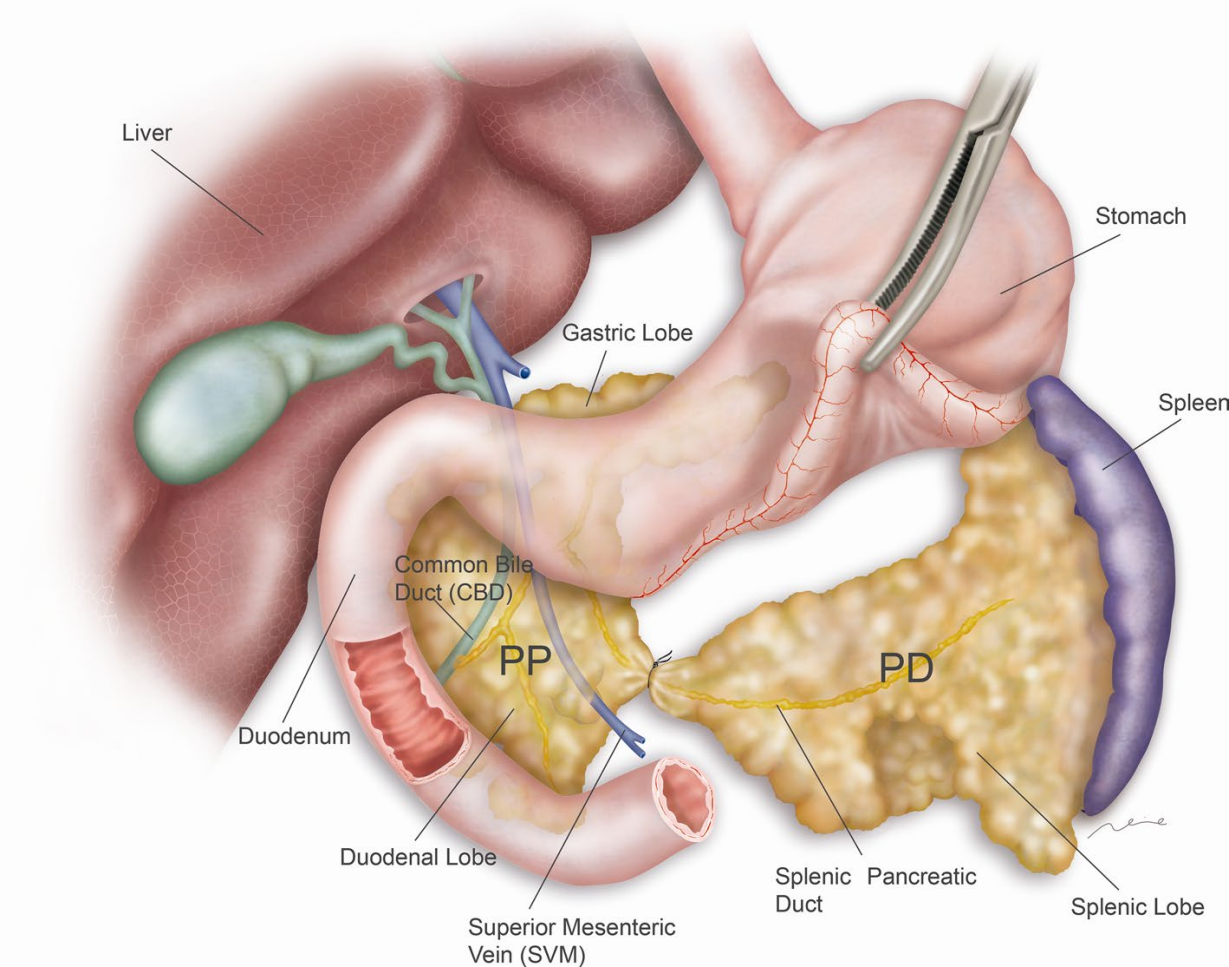
Surgical ligation of splenic pancreatic lobe in Ptf1a-Cre^(+/ki)^; K-ras LSLG12Vgeo^(+/ki)^ mice. Pancreatic ligation was performed including the main splenic pancreatic duct adjacent to the superior mesenteric vein. Two pancreatic segments, proximal pancreas (PP) and distal pancreas (DP) were thus delimited and separately analyzed.

At 12 weeks of age, the peritoneal cavity was accessed through a midline laparotomy, under anesthetic induction with isoflurane at 4% and at 2.5% maintenance in 100% oxygen. The stomach, pancreas and spleen were mobilized as described previously^19,24^ in all the animals subjected to surgery (sham and PDL groups). In PDL group mice, dissection and ligation of the splenic lobe of the pancreas were performed on the right side of the superior mesenteric vein, using 8/0 polyglycolic acid suture (Fig. 1)*.* Special caution with this technique was observed with the preservation of the gastroepiploic pedicle so that any ischemic effect on the pancreas and the spleen is avoided.

The pancreas and the abdominal viscera were finally relocated to their anatomical position, and the midline incision was sutured in two layers (aponeurotic plane with 4/0 absorbable continuous suture, and skin with 4/0 silk interrupted suture) in mice from the PDL and sham groups.

### Postoperative care and necropsy

**Figure 2:**
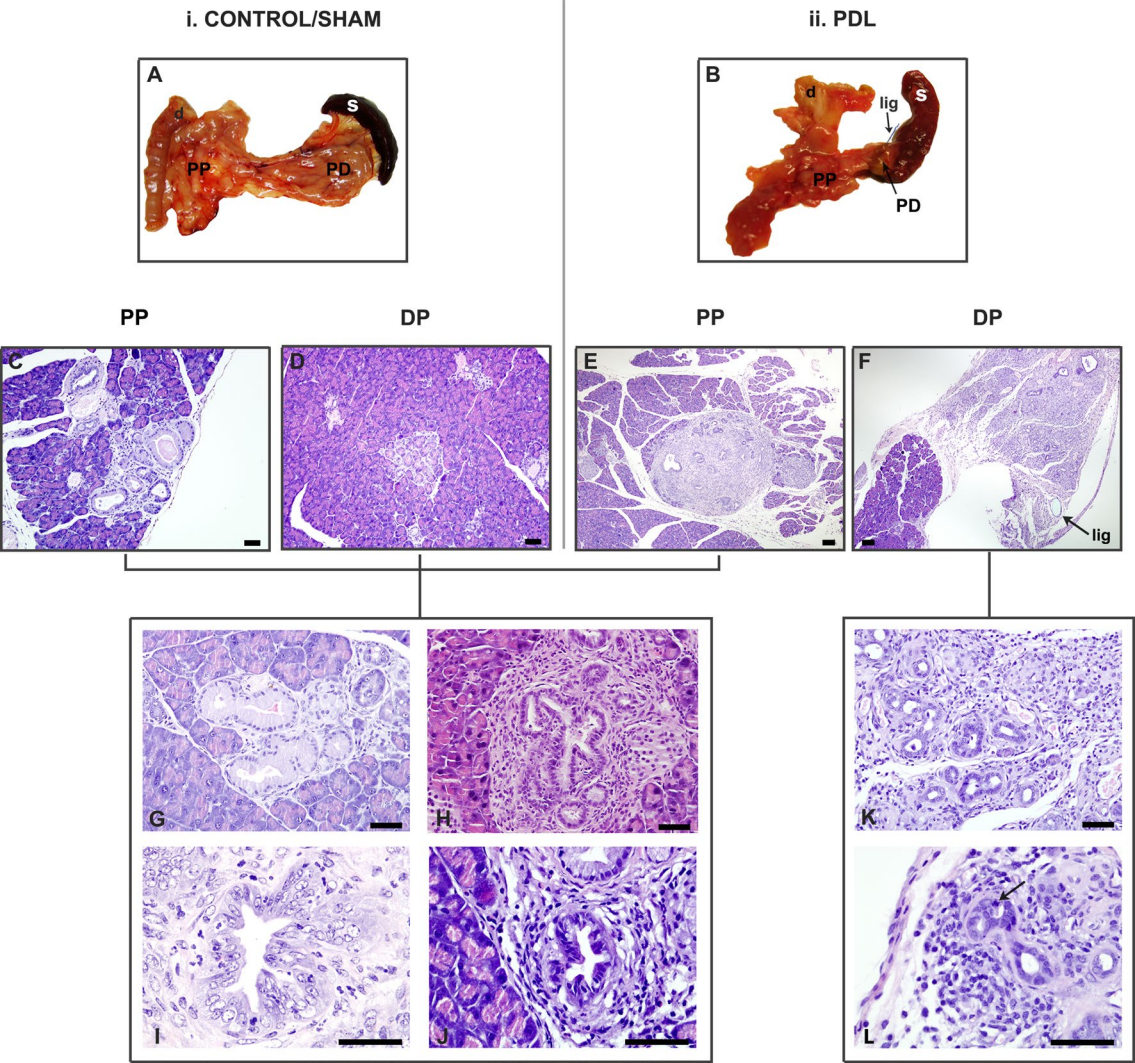
Histological aspects of PDL in Ptf1a-Cre^(+/ki)^; K-ras LSLG12Vgeo^(+/ki)^ mice. i. Macroscopic aspect of a Kras pancreas in a control or sham mice (**A**). Both proximal (**C**) and distal pancreas (**D**) developed PanIN1 (**G**), PanIN 2 (H), PanIN 3 (**I**) and AFL. (J). ii. Macroscopic aspect of a Kras pancreas of PDL mice (**B**). Note the atrophic aspect of the ligated distal pancreas which is shrunk to a small portion of the tissue close to the spleen (on the right of the ligature). Proximal pancreas (**E**) developed PanIN 1 (**G**), PanIN 2 (**H**), PanIN 3 (**I**) and AFL (**J**), while distal pancreas (**F**) was characterized by an atrophic pattern. Note preserved parenchyma before the ligature (arrow) and atrophic distal pancreas. Few PanIN 1 lesions and AFL were identified in the distal pancreas (**K**). But also an atrophic duct with an apoptotic cell (arrow), showing a pycnotic and irregular nucleus and surrounding the ducts moderate chronic inflammation (**L**). *d* duodenum, *s* spleen, *lig* ligature, *PP* proximal pancreas, *PD* distal pancreas. Scale bars (**C**–**D** and **G**–**L**): 50 μm. Scale bars (**E**–**F**): 200 μm.

Animals were monitored for signs of stress and followed up for 1, 3 or 6 months. At each of these PO periods, some mice of each group were euthanatized with CO_2_ inhalation to explore the peritoneal cavity. The pancreas, the spleen and a segment of the duodenum were simultaneously removed. The ligature, if present (see Fig. 2B,F), was noted and the splenic lobe was transected and referred as distal pancreas (DP) for further analysis. The other lobes (gastric and duodenal) were referred to as proximal pancreas (PP). The animals were euthanatized by inhalation of 70–90% CO_2_.

### Histological study

Specimens were formalin-fixed and embedded in paraffin. Each pancreas was serially sectioned (3 µm). A total of 11,200 slides were considered and a selection of 568 Sections (8 slides per animal, 70 mice in total) was stained with hematoxylin and eosin and reviewed blindly by an expert pathologist. Every preneoplasic lesion (PanIN 1 to PanIN 3 and AFLs), defined according to the 2006 Consensus^25^, was noted and counted^6,26^.

### Gene expression assays

Kras-PDL mice of different PO periods with enough quantity of atrophic distal pancreas to perform manual macroscopic dissection were selected for microarray analysis. The formalin-fixed, paraffin-embedded PP and DP of Kras-PDL mice were processed as previously described^27^. Briefly, total RNA was then isolated using the RecoverAll Total Nucleic Acid Isolation Kit (Life Technologies) from 40 serial sections with a thickness of 3 µm for each of the portions of the pancreas (PP and DP).

RNA quantity and purity was determined on the ND-2000 Spectrophotometer (NanoDrop Technologies) and RNA integrity was assessed using Agilent 2100 Bioanalyzer (Agilent Technologies). As RNA integrity number (RIN) obtained from Bioanalyzer is not informative for FFPE samples, the percentage of RNA > 200nt was used as an estimation of RNA integrity for each sample.

For microarrays analysis, amplification, labeling and hybridizations were performed according to protocol GeneChip Pico Reagent kit (Affymetrix). The working conditions used were 50 ng input RNA, 12 cycles of pre-PCR and 16 h of IVT. After the sample processing it was hybridized to GeneChip Mouse Clariom S Array (Affymetrix) in a GeneChip Hybridization Oven 640. Washing and scanning were performed using the Expression Wash and Stain and the GeneChip System of Affymetrix (GeneChip Fluidics Station 450 and GeneChip Scanner 3000 7G).

### Microarray data processing and gene expression analysis

Quality control was performed on the raw data CEL files corresponding to 5 paired Kras-PDL mice (DP and PP) samples with arrayQualityMetrics package^28^. Once quality checked, preprocessing of the data was executed with oligo package^29^. Normalization was performed using Robust Multi-array Average (RMA) algorithm which creates an expression matrix from Affymetrix data. The raw intensity values were background corrected, log2 transformed and then quantile normalized. Probes were annotated using annotateEset function and pd.clariom.s.mouse annotation data from the affycoretools package^30^. Next, a linear model was fit to the normalized data to obtain an expression measure for each probe set on each array. Before performing the differential gene expression analysis, the expression matrix obtained after RMA normalization was filtered by the Inter-quantile range (IQR) which provides a measure of the spread of the middle 50% of the intensity scores for each gene. Differential expression analysis was performed for paired samples using Limma^31^ (Linear Models for Microarray Data) package. Normalized expression matrix was used as input for Gene Set Enrichment Analysis (GSEA). Genesets with an FDR < 0.25 were considered significant.

### Human samples

Human pancreatic samples were obtained from patients who had undergone pancreatoduodenectomy (PD) with ductal occlusion of the remnant pancreas at the Parc de Salut Mar and had a primary diagnosis of PDAC or distal cholangiocarcinoma during the period 2012–2014. The protocol was approved by the Ethical Committee (CEIC-2012-130) of our institution (Hospital del Mar, Barcelona, Spain) and informed consent was obtained from all patients. All methods were performed in accordance with relevant guidelines and regulations for human subjects. In these selected cases, ductal occlusion of the main pancreatic duct of the distal remnant of the pancreas (without pancreatic anastomosis) was performed similarly the procedure described by Fromm et al.^32^ These patients provided a unique opportunity to study morphologic changes of the pancreas after pancreatic duct occlusion according to the evaluation of appearance on CT^33,34^ during the PO period. In addition, mutation analysis of exon 2, 3 and 4 of the KRAS gene was performed after manual or laser microdissection of precursor lesions in resected pancreas specimens.

### Statistical analysis

Data are presented as mean and standard deviation (SD). Non-parametric tests (Chi-Square, U-Mann Whitney and Kruskal–Wallis) were employed for univariate analysis. For exposing purposes, premalignant lesions were evaluated independently in PanIN 1 and higher grade premalignant lesions (HGPL) including PanIN 2, PanIN 3 and AFL. Linear regression models were performed in order to determine the best-fit equation for the number of lesions during follow-up for PanIN1 and HGPL. In addition, factors found to be significant predictors in the univariate analysis were subjected to multivariate analysis (backward stepwise logistic regression analysis). Results were expressed as Odds Ratio (OR) with a 95% confidence interval (CI). Differences were considered to be significant at a threshold of p < 0.05. Statistical analyses were performed with statistical software (SPSS version 19.0; SPSS, Inc, Chicago, Ill).

## Results

### Model validation

All animals tolerated the surgical procedure well and recovered and ambulated promptly. In the immediate PO period (4 days) the animals showed signs of stress and discomfort such as bristling hair or lack of grooming, as expected in a normal PO context. Later on, there were no signs of relevant disease in the PO follow-up and the Kras mice showed a normal growth curve, displaying no differences between groups.

As expected, premalignant lesions were not observed in control mice in which the conditional mutant Kras allele was not activated in the pancreas (Cre and G mice). In the Kras mice, we identified a majority of PanIN 1 lesions (n = 6290; 97%), followed in number by AFL (n = 160; 2.5%), PanIN 2 and PanIN 3 high-grade lesions (n = 32; 0.4% and n = 10; 0.1%, respectively) and frequent areas of focal lobulicentric atrophy (patchy atrophy) as already described in these models^26^. Therefore, hereinafter the results refer only to Kras mice. The time-course development of lesions is shown in Fig. 2.

Histologically, consistent with other PDL models^14,21,35^, the DP showed an intense pattern of atrophy with an increase of adipose and fibrosis over the PO periods. There also seemed to be an extension of adipose tissue surrounding the pancreatic tissue. More specifically, there was a complete replacement of exocrine acini by pseudoductal complexes, based on apparently novel duct-like structures composed of low cuboidal cells (Fig. 2F,K) (severe atrophy). There were also scattered infiltrates of lymphocytes and plasma cells in the fibrous boundary.

### The number of PanIN 1 lesions in the ligated pancreas is reduced

**Figure 3:**
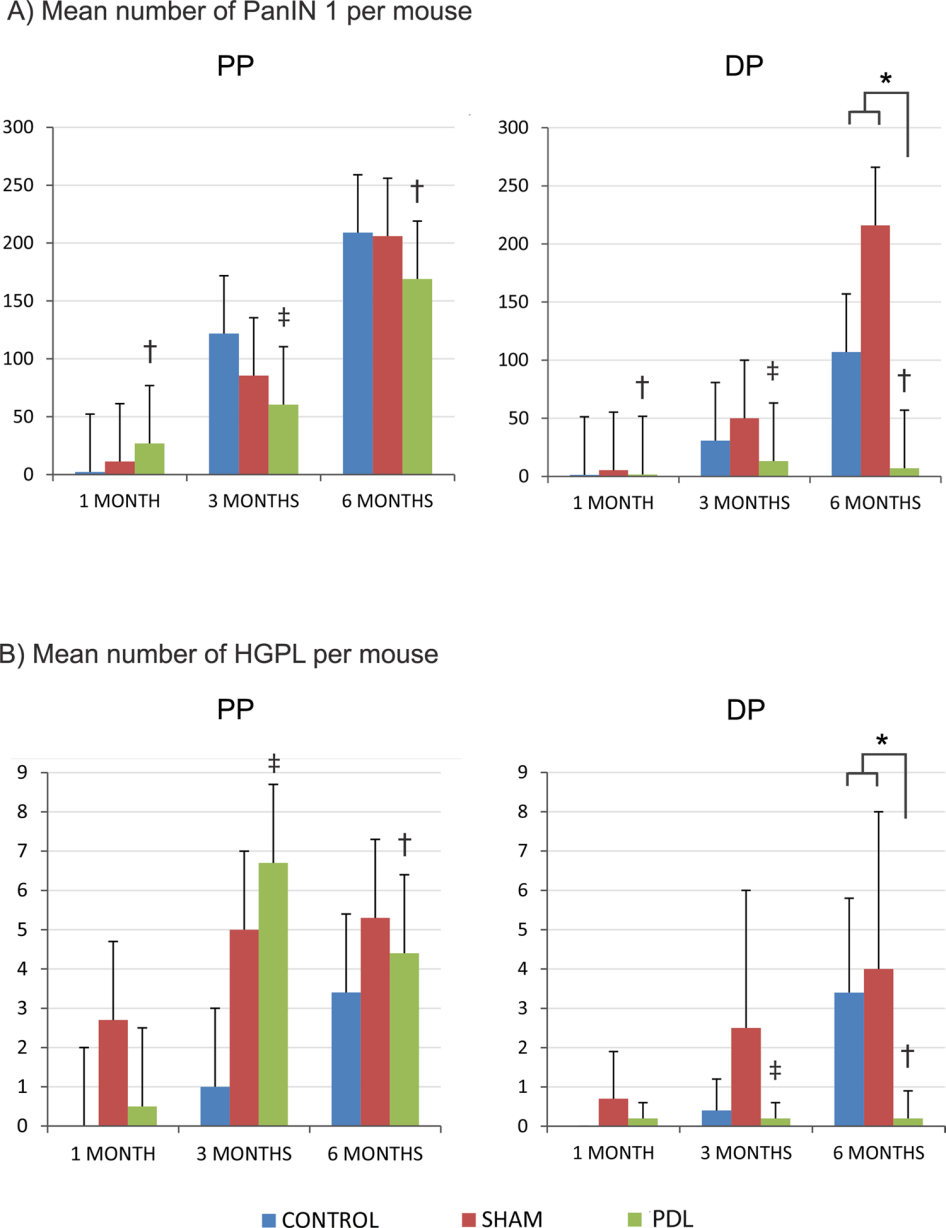
Evolution of preneoplasic lesions after PDL. Mean number of PanIN 1 (**A**) and HGPL (**B**) per mouse in each group over PO time. *Statistically significant (p < 0.05) comparing values between PDL vs. sham/control at DP after 6 month PO; ^†^Statistically significant (p < 0.05) comparing PP vs. DP for each group and PO period, ‡: p = 0.05.

All mice presented an increase of PanIN 1 lesions in the PP over time, without differences among groups (best-fit less squared function was obtained with a linear model; r = 0.57, p < 0.01). A similar increase was observed in the DP in the sham and control groups over the PO, but not in the PDL group. In fact, the number of PanIN 1 lesions in the PDL group was significantly lower in DP compared to its corresponding value in PP at each PO time being almost 24-fold lower at 6 months PO. Furthermore, the number of PanIN 1 lesion in DP was significantly lower (p < 0.01) in the PDL group at 6 months PO compared to the sham and control group (Fig. 3A).

### The number of HGPL in the ligated pancreas is reduced

Similarly to PanIN1 lesions, the number of HGPL in the PP usually increased over time in all groups and there were no significant differences between groups in any PO time. This increase was also observed in the DP of mice in the sham and control groups, which increased significantly over time following a linear model (r = 0.56, p < 0.01). Again, the number of HGPL in the PDL group was significantly lower in DP compared to its corresponding value in the PP at 3 and 6 months PO, being almost 22-fold lower at 6 months PO. Similarly to PanIN1 lesions, the number of HGPL in DP was significantly lower (p < 0.01) in the PDL group at 6 months PO compared to the sham and control group (Fig. 3B).

### The risk of developing HGPL is reduced in the ligated pancreas

In the univariant and multivariant analyses two variables demonstrated a significant influence on the presence of HGPL: postoperative time and experimental group. Thus, the increase of PO time was associated with 3 times more risk of HGPL (OR: 2.9, IC 95% 1.09–8.02, p = 0.03).

Having undergone PDL was protective for the development of HGPL: the risk of having HGPL was 6.87-fold higher in the control group (IC 95% 1.21–39.17, p = 0.03) and 9.84-fold higher in the sham group (IC 95% 1.21–785.92, p = 0.028) than in the PDL group.

**Figure 4:**
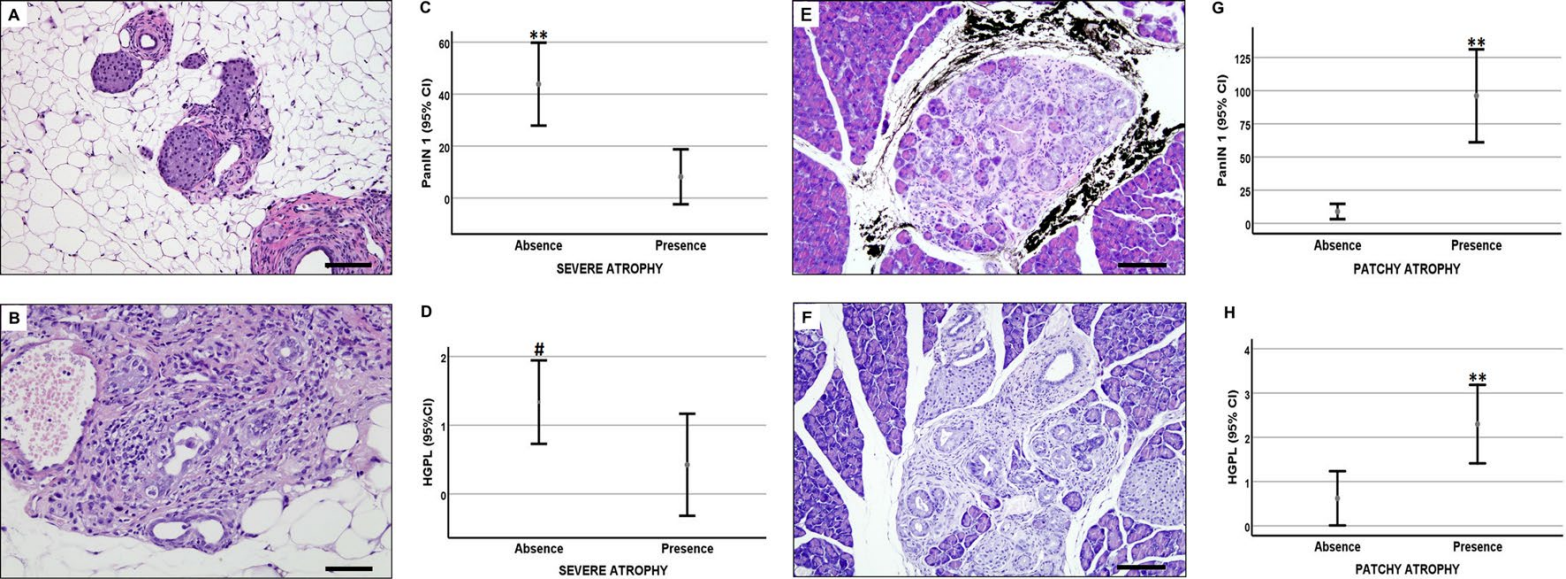
Types of atrophy in pancreas of Ptf1a-Cre^(+/ki)^; K-ras LSLG12Vgeo^(+/ki^ mice. When severe pancreatic atrophy (**A**.**B**) was present in the specimen (in DP of PDL-Kras mice) then PanIN 1 (**C**) and HGPL (**D**) were less frequently present than when there was no severe atrophy. On the contrary, when patchy atrophy (**E**,**F**) was observed in the specimen (in PP of control, sham and PDL-group and in DP of control and sham group) then PanIN 1 (**G**) and HGPL (**H**) were more frequently present than when patchy atrophy was absent. Bar errors including mean value and 95% confidence interval. *p < 0.05; **p < 0.01; ^#^p = 0.059. Scale bars: 50 μm.

A statistically significant decrease in the number of HGPL and PanIN 1 was observed when severe atrophy was seen in the studied segment of the pancreas (Fig. 4). On the contrary, patchy atrophy was linked to a significant increase in the number of HGPL and PanIN 1 (Fig. 4).

### Transcriptional analysis reveals downregulation of the acinar program in distal pancreas

**Figure 5:**
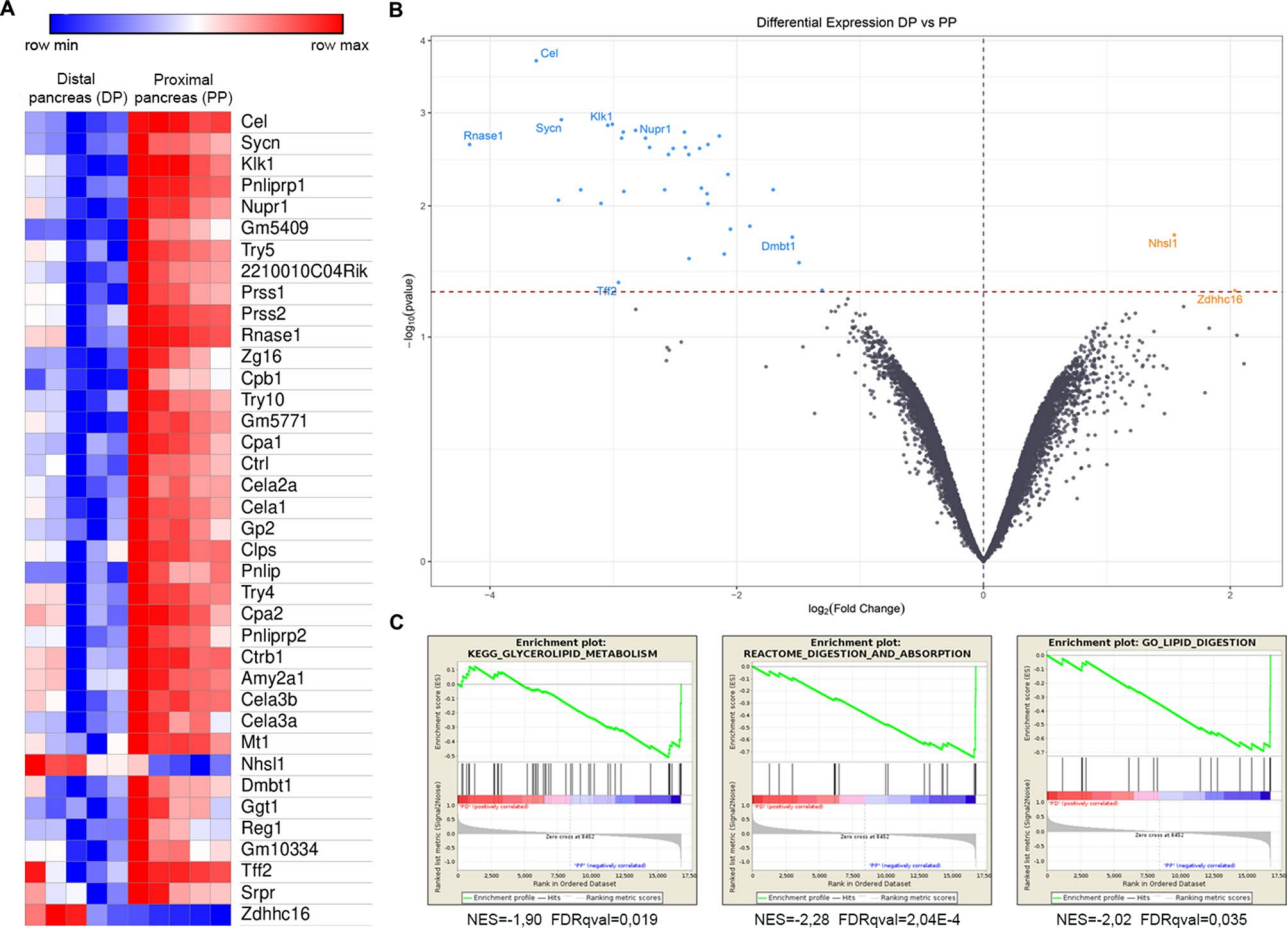
Differential gene expression after PDL. Hierarchical cluster heat-map of 38 differentially expressed genes between pancreatic segments (p-value < 0.05) (**A**). Volcano plot showing significative expressed genes, some of which are previously related to PDAC tumorigenesis (blue: upregulated in PP; yellow: upregulated in DP) (p-value < 0.05) (**B**). Gene Set Enrichment Analysis supports downregulation of the acinar function in the distal pancreas of Kras-PDL mice after ligation (p < 0.05) (**C**).

Differential expression analysis using Limma revealed 38 significant differentially expressed genes after p-value adjustment for multiple testing; 36 of those were downregulated and 2 upregulated in the distal portion of the ligated pancreas. The vast majority of downregulated genes corresponded to acinar enzymes or were related to the acinar function. (Table 1, Fig. 5A). Gene Set Enrichment analysis supported this downregulation of the acinar function in the distal pancreas of Kras-PDL mice. (Fig. 5B).

**Table 1:** Gene name, fold change and p-value of differentially expressed genes between PP and DP following PDL in Ptf1a-Cre^(+/ki)^; K-ras LSLG12Vgeo^(+/ki^ mice.

Gene symbol	Gene name	logFoldChange	adj.P.Val
Cel	Carboxyl ester lipase	− 3.63	0.0002
Sycn	Syncollin	− 3.42	0.0012
Klk1	Kallikrein 1	− 3.01	0.0014
Pnliprp1	Pancreatic lipase related protein 1	− 3.04	0.0014
Nupr1	Nuclear protein transcription regulator 1	− 2.82	0.0016
Gm5409	Predicted pseudogene 5409	− 2.42	0.0017
Try5	Trypsin 5	− 2.92	0.0017
2210010C04Rik	RIKEN cDNA 2210010C04 gene	− 2.14	0.0019
Prss1	Protease, serine 1 (trypsin 1)	− 2.74	0.0020
Prss2	Protease, serine 2	− 2.93	0.0020
Rnase1	Ribonuclease, RNase A family, 1 (pancreatic)	− 4.17	0.0024
Zg16	Zymogen granule protein 16	− 2.23	0.0024
Cpb1	Carboxypeptidase B1 (tissue)	− 2.71	0.0025
Try10	Trypsin 10	− 2.42	0.0025
Gm5771	Predicted gene 5771	− 2.30	0.0026
Cpa1	Carboxypeptidase A1, pancreatic	− 2.51	0.0026
Ctrl	Chymotrypsin-like	− 2.55	0.0030
Cela2a	Chymotrypsin-like elastase family, member 2A	− 2.39	0.0030
Cela1	Chymotrypsin-like elastase family, member 1	− 2.07	0.0049
Gp2	Glycoprotein 2 (zymogen granule membrane)	− 2.29	0.0068
Clps	Colipase, pancreatic	− 3.26	0.0070
Pnlip	Pancreatic lipase	− 1.70	0.0070
Try4	Trypsin 4	− 2.58	0.0070
Cpa2	Carboxypeptidase A2, pancreatic	− 2.91	0.0073
Pnliprp2	Pancreatic lipase-related protein 2	− 2.24	0.0077
Ctrb1	Chymotrypsinogen B1	− 3.45	0.0089
Amy2a1	Amylase 2a1	− 3.10	0.0095
Cela3b	Chymotrypsin-like elastase family, member 3B	− 2.23	0.0095
Cela3a	Chymotrypsin-like elastase family, member 3A	− 1.89	0.0152
Mt1	Metallothionein 1	− 2.05	0.0162
Nhsl1	NHS-like 1	1.55	0.0181
Dmbt1	Deleted in malignant brain tumors 1	− 1.55	0.0189
Ggt1	Gamma-glutamyltransferase 1	− 2.10	0.0260
Reg1	Regenerating islet-derived 1	− 2.39	0.0283
Gm10334	Predicted gene 10334	− 1.50	0.0304
Tff2	Trefoil factor 2 (spasmolytic protein 1)	− 2.96	0.0429
Srpr	Signal recognition particle receptor (docking protein)	− 1.31	0.0488
Zdhhc16	Zinc finger, DHHC domain containing 16	2.04	0.0491

38 genes were selected with a p-value < 0.05.

### Human experience in the ligated pancreas

**Figure 6:**
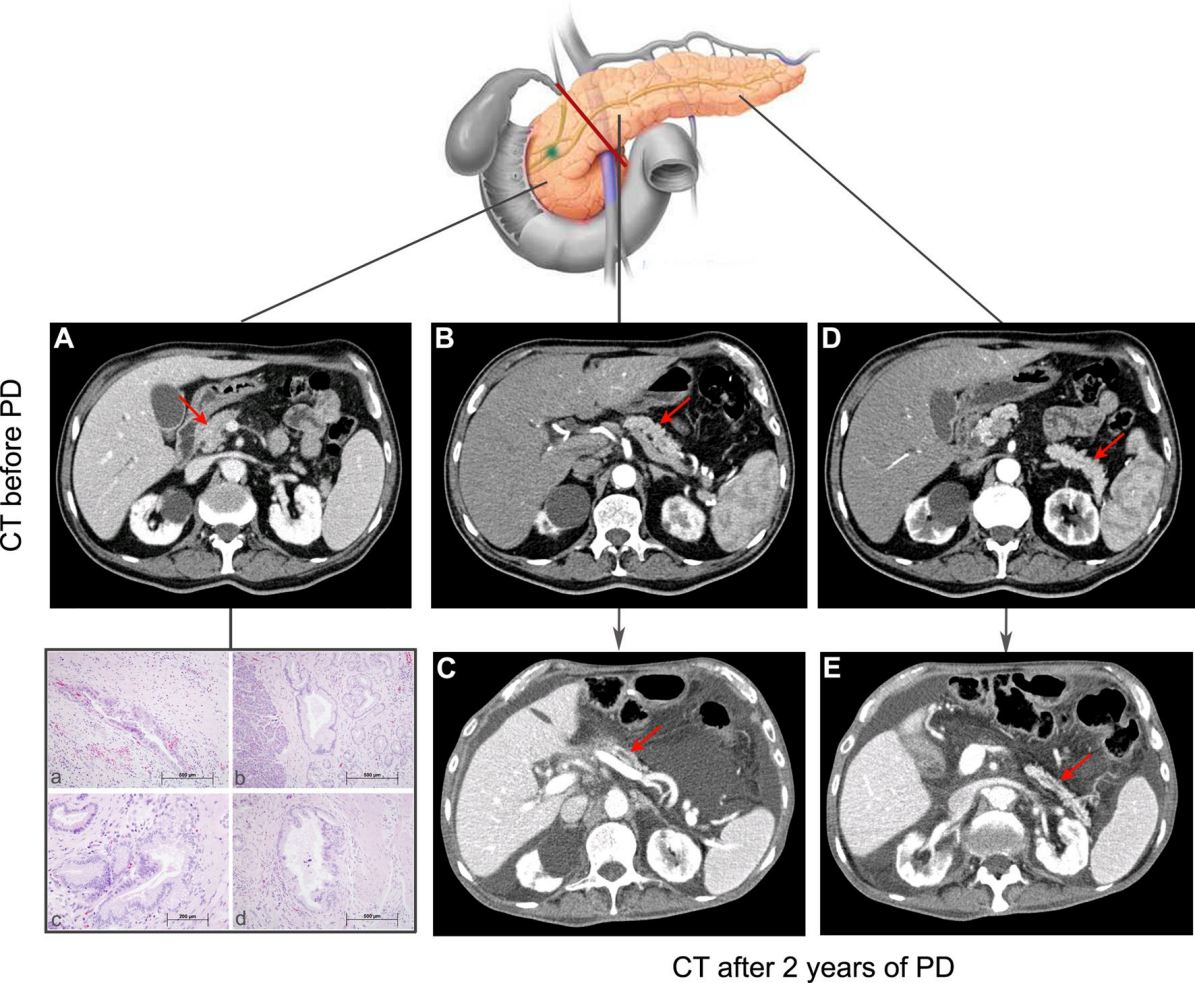
Human ligated pancreas. CT before pancreatoduodenectomy: head (**A**), body (**B**) and tail (**D**) of the pancreas (red arrows). CT after 2 years of the pancreatoduodenectomy (**C**,**E**). Note the reduction of the body (**C**) and tail (**E**) of the remaining pancreas. Hematoxylin–eosin stains of the resected head of the pancreas in which we can recognize the tumor (a), PanIN 1(b), PanIN 2 (c) and PanIN 3 (d).

Finally, to assess whether these effects were relevant to patients, we examined the pancreas of four patients with primary tumors in whom the distal pancreas was ligated after pancreatoduodenectomy (PD) that also contained PanIN1 and HGPL (see a representative case in Fig. 6). In all cases, the distal remnant pancreas (body and tail) was characterized by an atrophic pattern based on the CT analysis as it has been described in previous studies after PD^33,34^. The mean rate reduction of the pancreatic parenchymal thickness measured at the cross-section of its body after 12 and 24 months was 25 ± 15% and 47 ± 11%, respectively. An important ductal dilatation of the main pancreatic duct was also observed. In two of the cases exon 2 mutated to p.G12D and p.G12V. These observations are highly reminiscent of the findings in mice.

## Discussion

**Figure 7:**
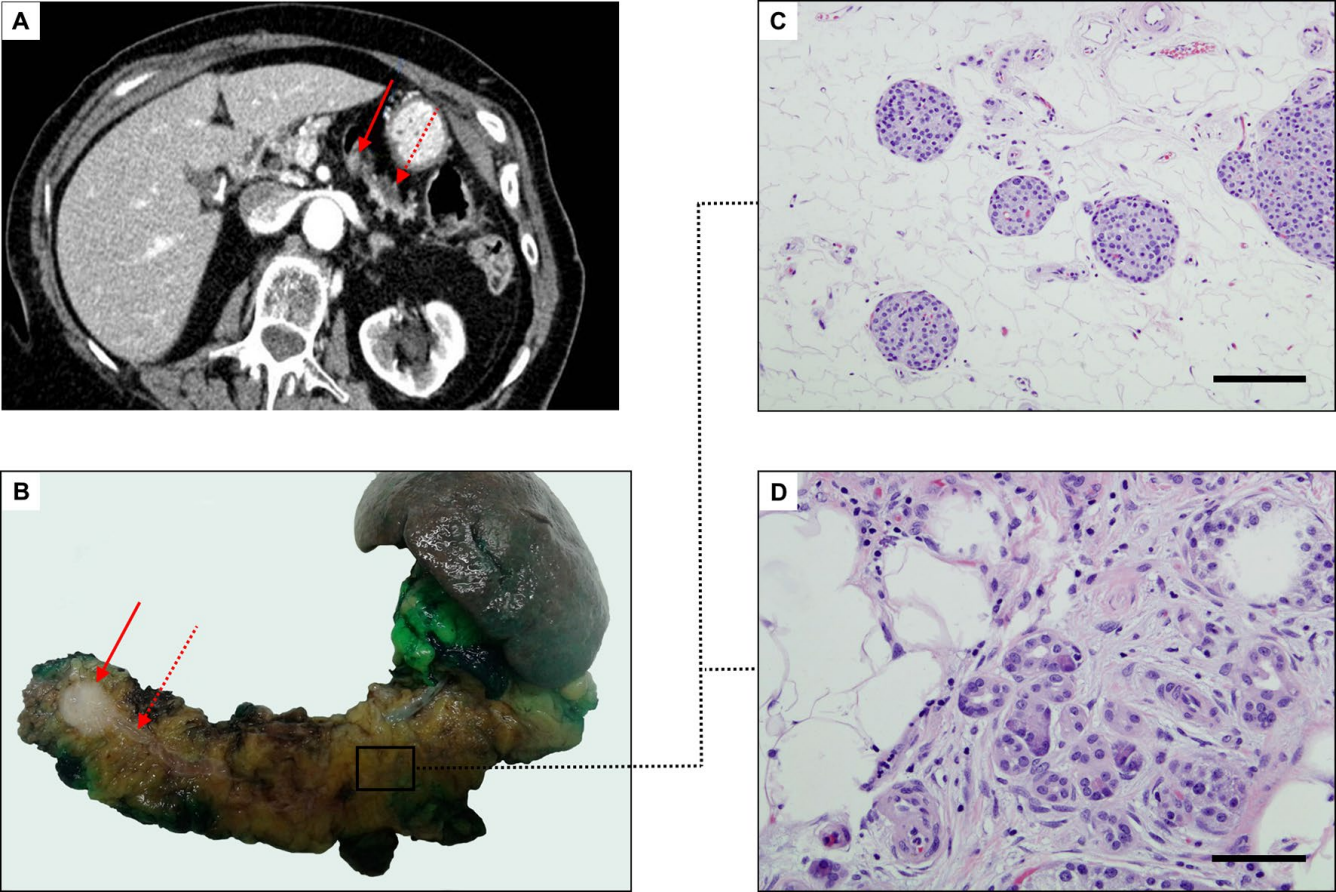
A resected human PDAC in the body of the pancreas that spontaneously lead to complete occlusion of the main pancreatic duct and complete atrophy of the distal pancreas. A female 84-year old patient was subjected to distal pancreatectomy with splenectomy for PDAC (pT3N0M0) in our institution. Preoperative CT imaging (**A**) as well as the surgical specimen (**B**) show the obstructive tumor, measuring 16 × 12 × 8 mm in the neck of the pancreas (arrow), and secondary dilation of the main pancreatic duct (dotted arrow). Note the severe atrophy of the distal pancreas (body and tail) at the histological level (**C**,**D**), where exocrine acini have been replaced by fibroadipose tissue, with preservation of endocrine Langerhans islets. Histhological images of atrophy in the distal pancreas are similar to the ligated distal pancreas in Kras mice (see Fig. 4). Scale bars: 50 μm.

Many conventional therapies for pancreatic cancer aim to induce cellular damage to trigger apoptosis. In fact, apoptosis is an evolutionary designed method of preventing propagations of mutations or simply to eliminate unwanted or useless cells^36^. Mutated or useless cells are better dead and cell suicide through apoptosis may achieve this goal and this could be relevant in many diseases. For example, in acute pancreatitis, the extent of pancreatic acinar cell apoptosis has been shown to be inversely related to the severity of the disease and induction of apoptosis in animal models has been found to have a protective effect against acute pancreatitis^37^. In other diseases like PDAC of the proximal pancreas, complete occlusion of the main pancreatic duct with the corresponding massive atrophy of the distal pancreas is frequent^38^ (see Fig. 7). In that regard, it has usually been recognized that complete occlusion of the main duct of exocrine glands leads to the deletion of acinar cells by a massive and rapid apoptosis phenomenon. This has been demonstrated in the pancreas^16–19^ and also in salivary glands^39^. We previously studied these phenomena with an efficient model of PDL in normal murine and normal pig pancreas in vivo^20,21^ and we confirmed a massive deletion of exocrine cells through apoptosis while preserving the endocrine tissue. In the present study we triggered the same physiological mechanism but in Kras mice at an early stage (when p53 is still active) in order to wipe out these potentially pro-oncogenic cells with a long period of follow-up (up to 6 months) in comparison to several intergroup controls (control and sham group) and intragroup controls (proximal pancreas). Very recently, two reports^40,41^ have described the influence of duct obstruction on the remaining distal pancreas in Kras mouse models at the cellular level but without quantitative analysis of preneoplastic lesions. Both of them described similar signs of strong atrophy and a complete loss of the acinar compartment with a ductal replacement but while Shi et al.^40^ described less preneoplastic lesions in the acinar differentiation models in the distal pancreas, Cheng et al.^41^ observed a higher number of proliferative non-mucinous cells of both acinar and ductal origins at 2 weeks after PDL, without severe nuclear atypia or tumoral infiltration after 5-month of follow up.

In our study, after a long follow-up (up to 6 months) and an exhaustive quantitative analysis, both the global evaluation of premalignant lesions (PanIN 1 and HGPL) and the risk of the apparition of them in a single animal were found to be dramatically lower in DP than in PP (more than 20 times lower) in PDL group and significantly lower than in sham and control groups.

Interestingly, animals with PDL experienced a marked reduction of the DP (severe atrophy) with preservation of the proximal one and this phenomenon was linked to a lower number of PanIN 1 and HPGL. In fact, the signs of severe atrophy of the distal pancreas after ligation were already evident through gross examination of the specimens at 1, 3 and 6 months PO (see the small remaining tissue of DP in Fig. 2B) and matched well with the corresponding microscopic examination of a complete acinar dropout. A similar atrophic pattern was seen spontaneously after occlusion of the main pancreatic duct by a proximal tumor in patients^38^ **(**Fig. 7) or over time in a very selective group of patients in which the remaining distal pancreas was ligated after pancreaticoduodenectomy based on the CT analysis (Fig. 6).

In the gene expression analysis of the animals, an expected reduction in pancreatic secretion was evident in DP samples of the PDL group. Interestingly, some top genes previously related to the development of preneoplastic lesions or PDAC had been upregulated in PP compared to DP like *Sycn*^42^, *Dmbt1*^43^*, Klk1*^44^ and mainly Nupr1. This last gene cooperates with KrasG12D to induce PanIN formation and pancreatic ductal adenocarcinoma development in mice^45–47^ (Fig. 5).

Our results are consistent with previous literature in which patchy atrophy accompanied by areas of acino-ductal metaplasia was constantly described in Kras models and usually linked to premalignant lesions^6,25,48,49^. Patchy atrophy has also been related to the presence of PanIN lesions in patients with familial predisposition to PDAC^6,14^. Acinar to ductal metaplasia has been associated with PanIN lesions and many acini in the lobules of lobulicentric atrophy described earlier are often characterized by prominent acinar-ductal metaplasia^14,26^. However, it has been serendipitously stated that when the atrophy is severe (> 90% loss of acini and ducts) is unusual to see PanIN lesions at least in the resected human pancreas without adenocarcinoma^50^. These findings are also in agreement with the currently accepted origin of PanIN lesions: observations indicate that the ductal-like cells present in PanINs rarely arise by the transformation of normal duct cells. Instead, PanINs may originate either by transdifferentiation of acinar cells or by misdifferentiation of their precursors^23,51,52^. Therefore, the transformation to PanIN lesions of these acinar cells or their precursors could be interrupted when an early obstruction of the main pancreatic duct is performed even when Kras pro-oncogenic stimulation is present. This could be a potential research line of interest in some cancerous risk patients taken into account that: (1) Complete occlusion of the main pancreatic duct could trigger a physiological protective effect to avoid malignancies in pancreas harboring Kras mutations without the aid of any other potentially dangerous treatments as it has been seen before; (2) This procedure could be feasible both surgically and endoscopically^53^; (3) Kras mutations are believed to be a common initiator of mucinous lesions and other cancer precursor lesions^54^ and (4) More than 90% of pancreatic adenocarcinomas harbor activating mutations in Kras for which targeted therapies have been notoriously ineffective across all cancer types prompting the perception that RAS is undruggable^54^. However, some limitations of this research must also be addressed: All the experiences were done in mice models in which early pancreatic lesions are evaluated after duct obstruction. Therefore, the final effect of duct obstruction on established PDAC especially in humans are beyond the scope of this study and could be detrimental for its evolution.

In conclusion, Pancreatic duct ligation in Ptf1a-Cre^(+/ki)^; K-ras LSLG12Vgeo^(+/ki)^ mice induces a decrease in the presence of premalignant lesions in the ligated distal pancreas. This could be a potential line of research of interest in some cancerous risk patients.

## Supplementary information

Supplementary Information.
