# Publisher Correction: Pancreatic duct ligation reduces premalignant pancreatic lesions in a Kras model of pancreatic adenocarcinoma in mice

**DOI:** 10.1038/s41598-021-85032-9

**Published:** 2021-03-02

**Authors:** Marta Cáceres, Rita Quesada, Mar Iglesias, Francisco X. Real, Maria Villamonte, Jaime Martinez de Villarreal, Mónica Pérez, Ana Andaluz, Xavier Moll, Enrique Berjano, Dimitri Dorcaratto, Patricia Sánchez-Velázquez, Luís Grande, Fernando Burdío

**Affiliations:** 1 Department of Surgery, Hospital del Mar, Barcelona, Spain; 2 Epithelial Carcinogenesis Group, Molecular Oncology Programme, Centro Nacional de Investigaciones Oncológicas, Madrid, Spain; 3 Departament de Medicina i Cirurgia Animals, Facultat de Veterinària, Universitat Autònoma de Barcelona, Barcelona, Spain; 4 BioMIT, Department of Electronic Engineering, Universitat Politècnica de València, Valencia, Spain; 5 Hospital Clínico de Valencia, Valencia, Spain

Correction to: *Scientific Reports*, 10.1038/s41598-020-74947-4, published online 27 October 2020

The original version of this Article contained a typographical error in the spelling of the author Patricia Sánchez-Velázquez, which was incorrectly given as Patricia Sánchez Velazquez. Additionally, the author Patricia Sánchez-Velázquez was incorrectly indexed.

These errors have now been corrected in the PDF and HTML versions of the Article.

