# Supplementary figures and images for: Pancreatic duct ligation reduces premalignant pancreatic lesions in a Kras model of pancreatic adenocarcinoma in mice
Source: Sci Rep. 2020 Oct 27;10:18344. doi: 10.1038/s41598-020-74947-4 (PMC7591874; doi:10.1038/s41598-020-74947-4)

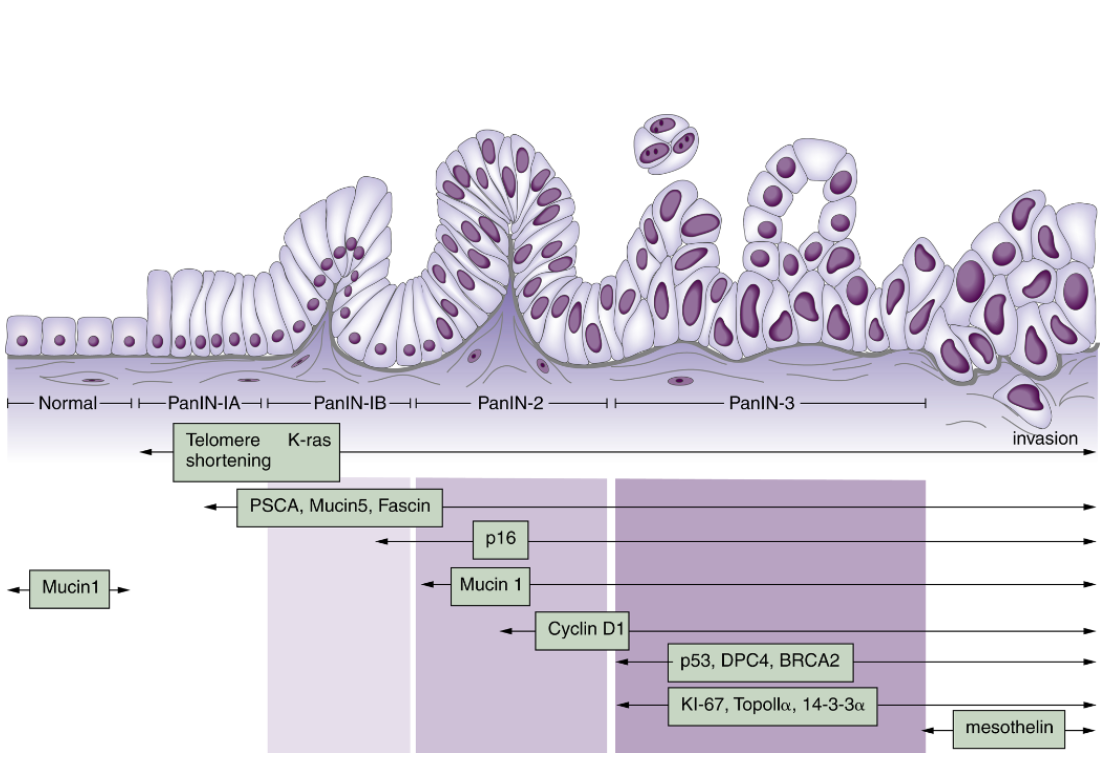

Supplement: Supplementary file 1 — Supplementary Information. [file 41598_2020_74947_MOESM1_ESM.tif]
